# Eomes Impedes Durable Response to Tumor Immunotherapy by Inhibiting Stemness, Tissue Residency, and Promoting the Dysfunctional State of Intratumoral CD8^+^ T Cells

**DOI:** 10.3389/fcell.2021.640224

**Published:** 2021-01-21

**Authors:** Runzi Sun, Yixian Wu, Huijun Zhou, Yanshi Wu, Zhongzhou Yang, Yanzheng Gu, Jingting Jiang, Binfeng Lu, Yibei Zhu

**Affiliations:** ^1^Department of Tumor Biological Treatment, The Third Affiliated Hospital of Soochow University, Changzhou, China; ^2^Department of Immunology, School of Biology and Basic Medical Sciences, Soochow University, Suzhou, China; ^3^Model Animal Research Center of Nanjing University, Nanjing, China; ^4^Department of Immunology, University of Pittsburgh School of Medicine, Pittsburgh, PA, United States; ^5^Jiangsu Key Laboratory of Clinical Immunology, Soochow University, Suzhou, China

**Keywords:** tumor immunotherapy, tissue residency, T cell dysfunction, stem-like T cell, tumor microenvironment

## Abstract

Sustaining efficacious T cell-mediated antitumor immune responses in the tumor tissues is the key to the success of cancer immunotherapy. Current strategies leverage altering the signals T cells sense in the tumor microenvironment (TME). Checkpoint inhibitor-based approaches block inhibitory signals such as PD-1 whereas cytokine-based therapies increase the level of immune-stimulatory cytokines such as IL-2. Besides extrinsic signals, the genetic circuit within T cells also participates in determining the nature and trajectory of antitumor immune responses. Here, we showed that efficacy of the IL33-based tumor immunotherapy was greatly enhanced in mice with T cell-specific Eomes deficiency. Mechanistically, we demonstrated that Eomes deficient mice had diminished proportions of exhausted/dysfunctional CD8^+^ T cells but increased percentages of tissue resident and stem-like CD8^+^ T cells in the TME. In addition, the IFNγ^+^TCF1^+^ CD8^+^ T cell subset was markedly increased in the Eomes deficient mice. We further demonstrated that Eomes bound directly to the transcription regulatory regions of exhaustion and tissue residency genes. In contrast to its role in inhibiting T cell immune responses at the tumor site, Eomes promoted generation of central memory T cells in the peripheral lymphoid system and memory recall responses against tumor growth at a distal tissue site. Finally, we showed that Eomes deficiency in T cells also resulted in increased efficacy of PD-1-blockade tumor immunotherapy. In all, our study indicates that Eomes plays a critical role in restricting prolonged T cell-mediated antitumor immune responses in the TME whereas promoting adaptive immunity in peripheral lymphoid organs.

## Introduction

IL33 is a member of the IL-1 gene family and highly expressed in epithelial cells of lining tissues (Moussion et al., 2008). Epithelial cell-expressed IL33 is associated with increased tumor immunogenicity by driving CD8^+^ T cell and dendritic cell type 1 (DC1)-dependent antitumor immunity (Lu et al., 2016; Chen et al., 2020). Consistently, IL33 down-regulation is associated with higher histological grade of various human carcinomas (Lu et al., 2016; Yang et al., 2018). Overexpression of active IL33 in tumor cells, administration of IL33 protein, as well as checkpoint inhibitor-induced tumor cell-derived IL33 inhibited tumor growth in multiple tumor models (Gao et al., 2015; Dominguez et al., 2017; Hollande et al., 2019; Chen et al., 2020; Moral et al., 2020). These studies support IL33 as a new immunotherapy for cancer. Despite strong antitumor efficacy, the genetic mechanism underlying CD8^+^ T cell-mediated immune responses in the IL33-impinged tumor microenvironment (TME) has not been explored.

Antitumor immunity is driven by both intra-tumoral and systemic tumor-antigen specific T cells. Many T cell functional subsets have been reported in the TME, including tissue resident T cells, effector T cells, exhausted/dysfunctional T cells, and stem-like T cells (Sakuishi et al., 2010; Gao et al., 2012; Guo et al., 2018; Kurtulus et al., 2019). The functional differentiation of CD8^+^ TIL subsets is regulated by an army of transcription factors such as Irf4, Batf, Nfatc1, and Tox for T cell exhaustion, Myb and TCF1 for “stemness,” and Bhlhe40, Runx3, Hobit, and Blimp1 for tissue residency (Mackay et al., 2016; Man et al., 2017; Milner et al., 2018; Gautam et al., 2019; Kurtulus et al., 2019; Li et al., 2019; Scott et al., 2019; Siddiqui et al., 2019). T-bet and Eomes, two members of T-box binding proteins, are considered master transcription factors regulating many CD8^+^ T cell subsets (Pearce et al., 2003; Szabo et al., 2015). T-bet and Eomes together were shown to be critical for trafficking of peripherally activated CD8^+^ T cells to the TME by induction of CXCR3 (Zhu et al., 2010). In addition, T-bet and Eomes are required for the generation of central memory T cells and inhibit the memory stem T cells (Intlekofer et al., 2005) (Li et al., 2013). During chronic infection, T-bet and Eomes are predominantly induced in effector and exhausted CD8^+^ T cells, respectively (Paley et al., 2012). Therefore, Eomes is suggested to play a role in T cell exhaustion (Doering et al., 2012; Paley et al., 2012; Li et al., 2018). Despite the extensive knowledge of Eomes immunobiology, the functional impact of Eomes on tumor immunotherapy is not well studied.

The success of tumor immunotherapy is strongly dependent on its impact on composition and functional states of CD8^+^ tumor infiltrating T cells (TIL). Because Eomes is a master transcription factor and has been implicated in regulating differentiation of many CD8^+^ T cell subsets, we set out to investigate the effect of Eomes on the IL33-based tumor immunotherapy. Our study is also designed to determine how Eomes shapes CD8^+^ T cell subsets in the TME.

## Results

**FIGURE 1:**
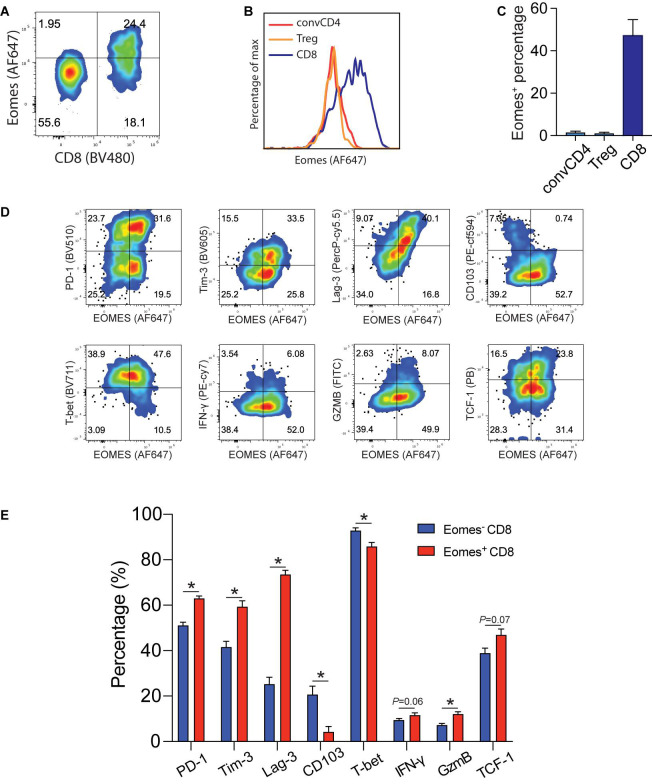
Eomes expression in B16-IL33 TILs. **(A)** Representative flow cytometry plot showing the percentage of Eomes expressing cells (gated on CD3^+^ T cells) in the B16-IL33 tumor. **(B,C)** Representative histograms **(B)** and corresponding quantification **(C)** of Eomes expressing conventional CD4^+^, Treg cells, and CD8^+^ T cells from the B16-IL33 tumor. **(D)** Representative flow cytometry plots showing Eomes co-stained with co-inhibitory molecules (PD-1, Tim-3, and Lag-3), effector molecules (IFN-γ, GzmB), tissue resident T cell markers CD103, and stem cell marker TCF-1 in CD8^+^ TILs. **(E)** Quantification of specific markers expression level in Eomes^–^ and Eomes^+^ CD8^+^ TIL shown in panel **(D)**, data were presented as mean ± SEM (*n* = 4). *P < 0.05, Student’s *t*-test was performed.

Tumor infiltrating Eomes^+^CD8^+^ T cells expressed higher levels of effector and exhaustion markers and lower levels of tissue residency markers. In order to understand the molecular underpinning of IL33-driven antitumor immunity, we examined Eomes expression in B16-IL33 tumor tissues. We found that Eomes mainly expressed in CD8^+^ T cells but not in conventional CD4^+^ T cells or Tregs (Figures 1A–C). We further characterized Eomes^+^ CD8^+^ TILs by multi-color flow cytometry (Figure 1D). We focused on four types of markers, namely co-inhibitory molecules, such as PD-1, Tim-3, and Lag-3, effector function markers, such as IFN-γ, and granzyme B, resident marker CD103, and resting marker TCF-1. We found that Eomes^+^ CD8^+^ TILs were composed of TCF1^+^ and TCF1^–^ subsets, suggesting Eomes is expressed in both activated and stem-like CD8^+^ T cells (Supplementary Figures 1A–D). We detected higher effector molecules such as IFN-γ, granzyme B and TNF-α in Eomes^+^ CD8^+^ TILs (Figure 1D and Supplementary Figure 1F), consistent with a functional role of Eomes in antitumor immunity (Li et al., 2013, 2018). We also found that Eomes^+^ CD8^+^ T cells had significantly higher expression of PD-1, Tim3, and Lag-3 compared with Eomes^–^ CD8^+^ T cells (Figure 1E), particularly in the Eomes^+^TCF1^–^ subset, suggesting Eomes play a role in T cell exhaustion (Supplementary Figures 1A–D). Interestingly, we observed that CD103 and Eomes expression was mutually exclusive, suggesting Eomes might inhibit CD103 expression in CD8^+^ TILs (Figures 1D,E). Taken together, these data suggested that Eomes might be involved in IL33-driven antitumor immune responses by affecting the function and generation of stem-like, effector, resident, and exhausted CD8^+^ TILs.

### Deletion of Eomes in T Cells Led to Inhibition of Tumor Growth

**FIGURE 2:**
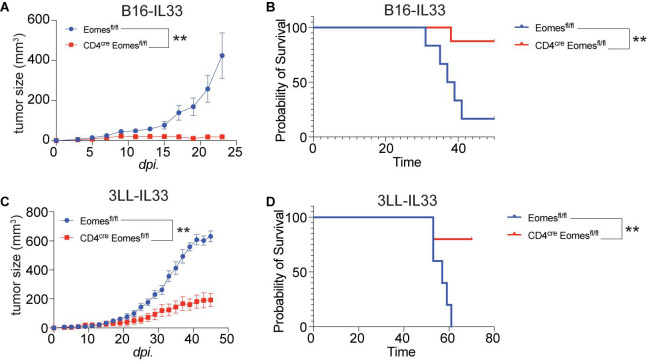
Deletion of Eomes in T cells resulted in arrest of growth of IL33-expressed tumors. **(A)** The B16-IL33 tumor cells (1 × 10^5^) were intradermally injected to control B6 or EKO B6 mice (*n* = 5–8, one of three independent experiments). Tumor sizes were monitored every 2 days, average sizes were shown. Two-way ANOVA was performed (**P < 0.01). **(B)** Overall survival of B16-IL33 tumor bearing mice in control and EKO mice. Log-rank test was performed (**P < 0.01). **(C)** The 3LL-IL33 tumor cells (2 × 10^5^) were intradermally injected to control B6 or EKO B6 mice (*n* = 5, one of two independent experiments). Tumor sizes were monitored every 2 days, average tumor sizes were shown. Two-way ANOVA was performed (**P < 0.01). **(D)** Overall survival of 3LL-IL33 tumor bearing mice in control and EKO mice. Log-rank test was performed (**P < 0.01).

To study the role of Eomes in IL33-driven antitumor immunity, we implanted B16 and B16-IL33 tumor cells into control and CD4^*cre*^ Eomes^*flox/flox*^ mice (EKO) (Figures 2A,B). B16 tumors grew at similar rate between control and EKO mice (Supplementary Figures 2A,B). B16-IL33 Tumor growth was initially comparable between control and EKO mice. However, despite expression of secreted IL33 in these tumors, tumor ultimately progressed rapidly in control mice (Figure 2A). In contrast, the tumor growth was arrested in EKO mice (Figure 2A), resulting in prolonged survival in EKO mice (Figure 2B). Besides B16 melanoma model, we also examined 3LL lung carcinoma to see whether this phenotype recapitulated in another tumor model. Similarly, there was no difference in growth rate and overall survival when transplanting wild type 3LL tumor (Supplementary Figures 2C,D). Also, we found 3LL-IL33 tumors grew at a significantly reduced rate (Figure 2C), and prolonged survival (Figure 2D) in EKO mice compared with control mice. These findings indicate that Eomes impedes the sustained antitumor efficacy of IL33.

### Eomes Deficiency Resulted in Increased Tumor Infiltrating Lymphocytes

**FIGURE 3:**
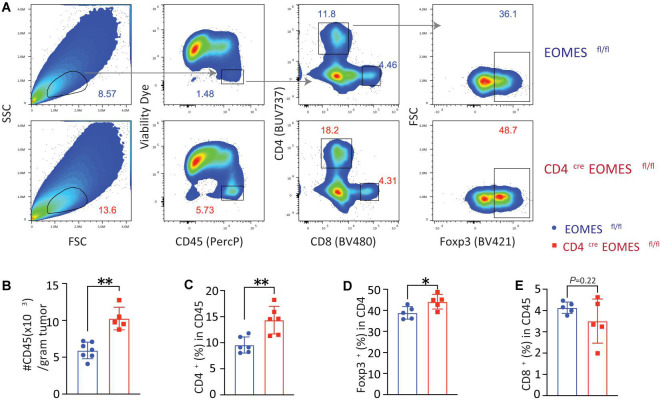
Eomes deletion in T cells led to increased tumor infiltrating lymphocytes. **(A)** The B16-IL33 tumor cells (1 × 10^5^) were intradermally injected to control or EKO mice. 17 days after tumor inoculation, tumors were resected and analyzed by flow cytometry. Representative flow cytometry plots depicting the gating strategy of main immune populations. **(B)** Quantification of the number of CD45^+^ tumor infiltrating lymphocytes per gram tumor tissue. **P < 0.01, Student’s *t* test was performed. **(C)** Quantification of the percentage (CD45 gated) of CD4^+^ T cells. **P < 0.01, Student’s *t* test was performed. **(D)** Quantification of the percentage (CD4 gated) of Foxp3^+^ Treg cells. *P < 0.05, Student’s *t*-test was performed. **(E)** Quantification of the percentage (CD45 gated) of CD8^+^ T cells. Student’s *t* test was performed.

In order to better understand the underling immune mechanisms that are responsible for heightened antitumor activities in EKO mice, we studied immune cells in the tumor (Figure 3). Compared with tumors harvested from control mice, we saw a twofold increase in the CD45^+^ immune cells in tumors isolated from EKO mice (Figures 3A,B). Within the CD45^+^ immune cells, we found that the percentages of CD4^+^ T and Foxp3^+^ Treg cells were higher in the EKO mice (Figures 3C,D). In contrast, we found no significant difference in the percentage of CD8^+^ T cells between tumors in control and EKO mice (Figure 3E). Given the increase in the number of infiltrated lymphocytes, the number of CD8^+^ TILs was still higher in tumors from EKO tumors. Overall, these results indicate that Eomes deficiency resulted in more inflamed tumors.

### Eomes Deficiency in T Cells Led to Increased Stem-Like and Tissue Resident CD8^+^ TILs

**FIGURE 4:**
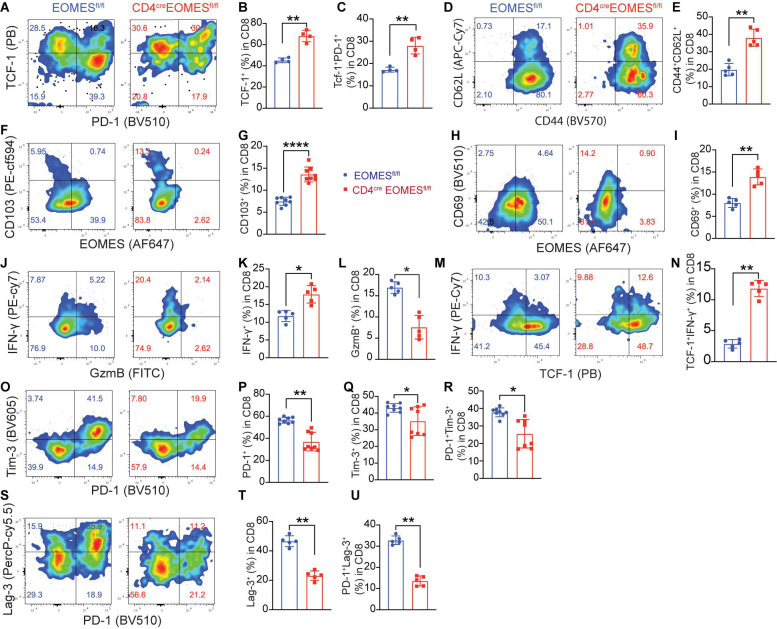
Deletion of Eomes in T cells altered TME. **(A)** The B16-IL33 tumor cells (1 × 10^5^) were intradermally injected to CON or EKO mice. 17 days after tumor inoculation, tumors were resected and analyzed by flow cytometry. Representative flow cytometry plots showing PD-1 and TCF-1 staining in CD8^+^ T cells. **(B,C)** Percentage of TCF-1^+^CD8^+^ T cells **(B)** and TCF-1^+^PD-1^+^CD8^+^ T cells **(C)** were shown. **P < 0.01, Student’s *t*-test was performed. **(D,E)** Representative flow plots **(D)** and corresponding quantification **(E)** of CD62L (L-sel) and CD44 in CD8^+^ T cells. **P < 0.01, Student’s *t*-test was performed. **(F–I)** Representative flow cytometry plots depicting the CD103 **(F)** and CD69 **(H)** expression gated on CD8^+^ T cells. Quantification of the percentage of CD103 **(G)** and CD69 **(I)** expressing cells in CD8^+^ TILs. Data were presented as mean ± SEM. ****P < 0.0001, Student’s *t*-test was performed. **(J)** Representative flow cytometry plots showing IFN-γ and GzmB production by tumor infiltrating CD8^+^ T cells. **(K-L)** Quantification of the percentage of IFN-γ^+^ CD8^+^ T cells **(K)** and GzmB^+^ CD8^+^ T cells **(L)**. *P < 0.05, Student’s *t*-test was performed. **(M)** Representative flow cytometry plot showing IFN-γ and TCF-1 staining between control and EKO tumors. **(N)** Quantification of the percentage of TCF-1^+^IFN-γ^+^ in CD8^+^ T cells. **P < 0.01, Student’s t test was performed. **(O)** Representative flow cytometry plots depicting the PD-1 and Tim-3 expression pattern in the tumor infiltrating CD8^+^ T cells. **(P–R)** Quantification of the percentage of PD-1^+^, Tim-3^+^, and PD-1^+^TIM-3^+^ in CD8^+^ T cells. Data were presented as mean ± SEM. *P < 0.05, **P < 0.01, Student’s *t*-test was performed. **(S)** Representative flow cytometry plots depicting the PD-1 and Lag-3 expression pattern in the tumor infiltrating CD8^+^ T cells. **(T–U)** Quantification of the percentage of Lag-3^+^, and PD-1^+^Lag-3^+^ in CD8^+^ T cells. **P < 0.01, Student’s *t*-test was performed.

Because Eomes is predominantly expressed in CD8^+^ TILs, we examined impact of Eomes deletion on major CD8^+^ T cell subsets in the TME including stem-like, effector, tissue resident, and exhausted TILs. Stem-like CD8^+^ T cells are characterized by expression of TCF-1 and CD62L (Yu and Anasetti, 2005; Gattinoni et al., 2009; Thommen, 2019). We found that both TCF-1^+^CD8^+^ T cells were significantly increased in B16-IL33 tumors isolated from EKO mice (Figures 4A,B) compared to control mice. Consistent with this finding, we also found that CD62L^+^ CD8^+^ T cells were increased in tumors from EKO mice compared to WT mice (Figures 4D,E). A population of PD-1^+^TCF1^+^CD8^+^ T cells were found in chronic infection and TME and is called precursor exhausted T cells (Kurtulus et al., 2019). We found that TCF-1^+^PD-1^+^ CD8^+^ subset was also increased in EKO TME (Figures 4A,C). These data suggest that Eomes inhibits the frequency of stem-like CD8^+^ T cells in the IL33-shaped TME.

Since we recently reported that IL33 promoted the accumulation of tissue resident T cells in the TME (Chen et al., 2020), we decided to examine whether Eomes deficiency affected the portion of resident CD8^+^ T cells. We found the tissue resident markers CD103 and CD69 were significantly higher in EKO CD8^+^ T cells in B16-IL33 tumors from EKO mice than control mice (Figures 4F–I), indicating that Eomes inhibited the generation of resident CD8^+^ T cells in the TME. Collectively, these results illustrated a critical role of Eomes in inhibiting stem-like and tissue resident CD8^+^ TILs.

### Eomes Deficiency Led to Increased IFN-γ in Stem-Like TCF1^+^ CD8^+^ TIL but Decreased GzmB in TCF1^–^CD8^+^ TILs

We next examined T cell effector molecules, such as IFN-γ and GzmB, in CD8^+^ TILs from WT and EKO mice. Our analysis showed that the percentage of IFN-γ^+^CD8^+^ TILs was greatly increased in B16-IL33 tumors from EKO mice compared to WT mice (Figures 4J,K). Interestingly, we found the increased IFN-γ^+^ CD8^+^ TILs in EKO were mainly in the stem-like T cell compartment (Figures 4M,N), suggesting Eomes inhibits IFN-γ^+^ exclusively in the stem-like CD8^+^ T cell compartment. In contrast, the percentage of GzmB^+^CD8^+^ TILs was profoundly decreased in EKO mice compared to WT mice (Figures 4J,L), consistent with prior publications indicating that Eomes directly regulates cytolytic genes (Pearce et al., 2003). GzmB was only expressed in TCF1^–^ CD8^+^ TILs (Supplementary Figures 3A,B). These results suggest that Eomes regulates IFN-γ^+^ and GzmB in different CD8^+^ TIL subsets. These data are also in line with our pervious results demonstrating that IFN-γ but not perforin is required for the antitumor function of IL33 (Gao et al., 2015).

### Eomes Deficiency in T Cells Led to a Decrease in Exhaustion/Dysfunction Markers in CD8^+^ TIL Subsets

Exhausted T cells are a type of hyper activated effector cells characterized by expression of co-inhibitory receptors including PD-1, Tim-3, and Lag-3 (Fourcade et al., 2010; Sakuishi et al., 2010; Wherry, 2011; Gao et al., 2012; Yang et al., 2020). Eomes has been associated with T cell exhaustion state (Doering et al., 2012; Paley et al., 2012; Li et al., 2018). Therefore, we studied the expression of co-inhibitory receptors in CD8^+^ TILs using flow cytometry and found that percentages of PD-1^+^, Tim-3^+^, Lag-3^+^, PD-1^+^Tim3^+^, and PD-1^+^Lag-3^+^ cells were significantly reduced in B16-IL33 tumors from EKO mice than control mice (Figures 4O–U). Tim-3^+^ CD8^+^ TILs were mainly TCF-1^–^ (Supplementary Figures 3C–F), consistent with a role of Eomes in T cell exhaustion. In contrast, Lag3^+^ was present on both TCF-1^+^ and TCF-1^–^ CD8^+^ TILs. Eomes deficiency resulted in a reduction of Lag3 in both TCF-1^+^ and TCF-1^–^ CD8^+^ TIL (Supplementary Figures 3C–F), suggesting Eomes inhibits the function of stem-like CD8^+^ T cells through Lag3. These data indicate that Eomes is required for the upregulation of multiple co-inhibitors in CD8^+^ TILs.

### Eomes Directly Inhibited Expression of CD103 and IFN-γ

**FIGURE 5:**
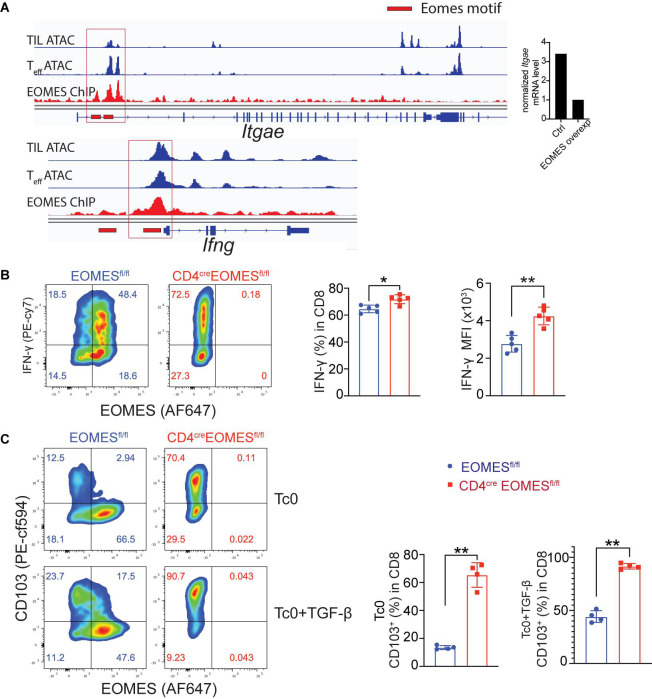
Eomes regulated CD103 and IFN-γ *in vitro*. **(A)** Previously published Eomes-ChIPseq tracks (GSE122895), ATACseq tracks of tumor infiltrating CD8^+^ T cells (GSE110251) and *in vitro* cultured effector CD8^+^ T cells (GSE86797) were integrated in IGV genome browser at the CD103 and IFN-γ loci with Eomes binding peaks (red lines). The normalized CD103 mRNA level between control and Eomes-overexpressed CD8^+^ T cells measured by RNA-seq (GSE122895) was also shown (right part). **(B,C)** Total CD8^+^ T cells were purified from spleen and lymph nodes of control or EKO mice and cultured under Tc0 condition for 4 days, and then either stimulated with TGF-β for CD103 induction or with IL12 and IL33 for IFN-γ induction for 24 h. Representative flow cytometry plots and quantification of the percentage of IFN-γ^+^ CD8^+^ T cells **(B)** and CD103^+^CD8^+^ T cells **(C)**. Data were presented as mean ± SEM. *P < 0.05, **P < 0.01, Student’s *t*-test was performed.

Prompted by observation of increased IFN-γ and resident marker CD103 in EKO CD8^+^ TILs, we asked whether Eomes, as a transcriptional factor, directly regulated CD103 and IFN-γ gene transcription in CD8^+^ T cells. To that end, we integrated data from multiple genetic platforms and aligned an Eomes ChIP-seq track generated from Eomes-overexpressing OT-I CD8^+^ T cells, an ATAC-seq track generated from *in vitro* cultured effector CD8^+^ T cells, and an ATAC-seq track generated from CD8^+^ TILs. We then determined whether Eomes bound to the promoter regions of the key marker genes that we saw differences *in vivo*. We found that Eomes bound directly around the promoter region of both *Itgae* and *Ifng* gene loci (Figure 5A). In addition, we showed that both the percentage of IFN-γ^+^ cells and the mean fluorescence intensity (MFI) of IFN-γ protein were higher in EKO CD8^+^ T cells compared to WT CD8^+^ T cells (Figure 5B). TGF-β was reported to induce the expression of CD103 (El-Asady et al., 2005). We showed that Eomes deficiency resulted in increased CD103 expression when CD8^+^ T cells were cultured with or without TGF-β (Figure 5C). In the same vein, we also showed that Eomes bound directly to regulatory regions in genes encoding exhaustion markers including PD-1, Tim-3, and Lag3 (Supplementary Figure 4). Taken together, our data suggested that Eomes directly regulated transcription of CD103, IFN-γ, and exhaustion marker genes.

### Eomes Deficiency in T Cells Led to Diminished Peripheral Memory Antitumor Immune Responses

**FIGURE 6:**
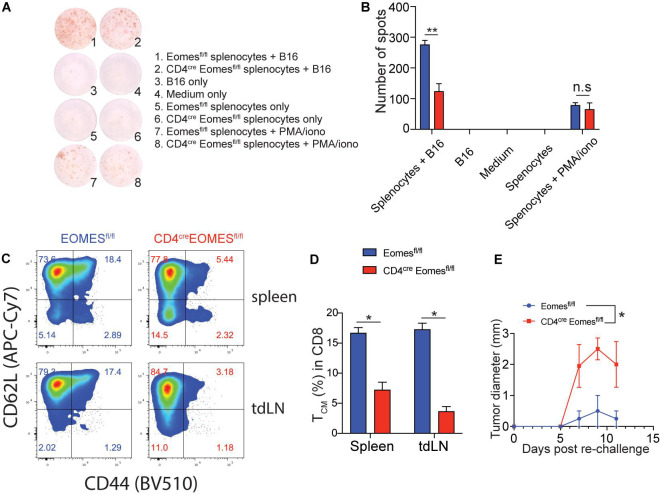
The peripheral tumor antigen specific immune response was impaired in EKO mice. **(A,B)** B16-IL33 cells were intradermally injected to control and EKO mice. 17 days after tumor inoculation, immune cells from spleen and tumor draining lymph nodes were analyzed. IFN-γ ELISpot assay was performed using splenocytes. representative ELISpot pictures **(A)** and corresponding quantification of IFN-γ spots **(B)** were shown. Data were presented as mean ± SEM. **P < 0.01, Student’s *t*-test was performed. **(C–E)** Flow cytometry analysis was carried out for memory markers CD44 and CD62L on total CD8^+^ T cells. Representative flow plots **(C)** and corresponding quantification **(D)** of central memory T cells among CD8^+^ T cells were shown. *P < 0.05, Student’s *t*-test was performed. **(E)** The B16-IL33 tumor cells (1 × 10^5^) were intradermally injected to the right flank of control and EKO mice. 17 days after initial tumor inoculation, B16 tumor cells (1 × 10^5^) were injected to the left flank of the tumor bearing mice. Tumor sizes were monitored every 2 days, average tumor sizes were shown. Two-way ANOVA was performed (*P < 0.05).

Our current data suggest that Eomes inhibits T cell-mediated immune responses in the TME in the setting of IL33-based tumor immunotherapy. It remained to be evaluated the role of Eomes in systemic antitumor immunity in the same setting. To address this question, we performed the enzyme-linked immune absorbent spot (ELISpot) assay for IFN-γ to assess the systemic tumor antigen specific response in WT and EKO mice (Figures 6A,B). The frequency of tumor antigen specific IFN-γ-producers was much lower in the splenocytes from EKO mice than those from control mice, indicating Eomes is required for systematic T cell immune responses. In addition, our data showed that the percentage of total CD44^+^CD62L^+^ central memory CD8^+^ T cells (T_*cm*_) was significantly diminished in EKO mice compared to control mice in both spleens and tumor draining lymph nodes (Figures 6C,D). This result contrasted strikingly with the increased CD62L^+^ CD44^+^CD8^+^ T_*cm*_ cells in TME (Figures 4D,E) suggesting Eomes regulates different sets of genes in the tumor tissues and the second lymphoid system. We then examined the memory recall immune responses by inoculating the same tumor cells on another site of the same tumor-bearing mice. We found that both WT and EKO mice generated immune responses and tumor grew but eventually stabilized. However, the tumor grew to a larger size in EKO mice compared to WT mice (Figure 6E). Collectively, these data demonstrated Eomes is required for T cell memory immune responses.

### Eomes Deficiency in T Cells Greatly Enhanced PD-1-Blockade Tumor Immunotherapy

**FIGURE 7:**
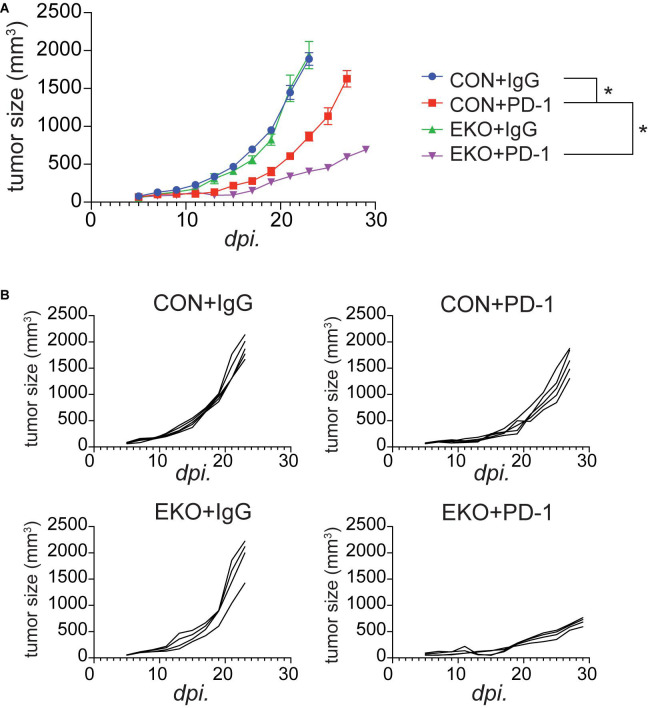
Deletion of Eomes in T cells combined with PD-1 further inhibited tumor growth. **(A)** The MC38 tumor cells (1 × 10^6^) were intradermally injected to control B6 or EKO B6 mice. Tumor sizes were monitored every 2 days, average sizes were shown. Two-way ANOVA was performed (*P < 0.05). **(B)** Individual tumor curves of mice depicted in **(A)**.

We recently showed that IL33 was induced by PD-1 monoclonal antibodies (mAbs) in tumor cells and was crucial for the antitumor effect of this checkpoint inhibitor in mouse tumor models (Chen et al., 2020). The finding of an important role of Eomes in perpetuating antitumor efficacy of IL33 prompted us to investigate whether Eomes deficiency also affected the therapeutic effect of PD-1 mAbs. We inoculated MC38 tumor cells in WT and EKO mice and administered PD-1 mAbs. As expected, administration of PD-1 mAbs inhibited tumor growth (Figures 7A,B). Strikingly, Eomes deficiency led to much slower tumor growth compared to WT control mice (Figures 7A,B). Therefore, Eomes expression in T cells significantly limits the therapeutic efficacy of both checkpoint inhibitor- and cytokine-based tumor immunotherapies.

## Discussion

This study has established a critical role of Eomes in limiting sustained efficacy of IL33-based and PD-1 blockade cancer immunotherapy. Mechanistically, Eomes inhibits T cell-mediated antitumor activities by enhancing T cell exhaustion, reducing the number and IFN-γ production by stem-like CD8^+^ T cells, and inhibiting tissue residency molecules. Our data also indicates that the Eomes’ inhibitory function is restricted to the tissue level. In the peripheral lymphoid system, in contrast, Eomes promotes adaptive antitumor immunity by enhancing memory T cells. Our results illustrate a critical biological function of Eomes in resolving tissue-level T-cell activities and promoting systemic adaptive immunity.

Our study solidifies a crucial role of Eomes in inhibiting T cell immunity in tumor tissues during sustained T cell-mediated antitumor immune responses by regulating the differentiation and function of multiple CD8^+^ TIL subsets. Eomes was shown to be predominantly expressed in exhausted CD8^+^ T cells in a chronic infection model, suggesting a role in T cell exhaustion (Paley et al., 2012). Nevertheless, Eomes deficiency did not result in heightened antiviral responses. In fact, deletion of Eomes leads to impaired maintenance of the antiviral CD8^+^ T cell response and increased viral titer (Paley et al., 2012). Another study showed that deletion of one allele of Eomes in T cells diminished exhausted CD8^+^ T cells and showed better tumor control. However, the same study also showed that complete loss of Eomes in T cells resulted in impaired development of anti-tumor immunity (Li et al., 2018). These discrepancies in the literature about the functional consequences of Eomes deficiency need to be resolved (Li et al., 2018; Weulersse et al., 2020). Our data clearly demonstrated that Eomes deficiency greatly enhanced antitumor activities and increased the durability of IL33 and checkpoint blockade tumor therapy models. The profound functional impact of Eomes in our model reflected a critical role of Eomes in IL33-driven hyper activated T cell-predominant TME. In this setting, one major function of Eomes is to diminish exuberant T cell immune responses by promoting the genetic program of exhaustion/dysfunction. In addition, it is noted that IFN-γ is increased in the intratumoral CD8^+^ T cells of EKO mice. IFN-γ is the pivotal cytokine anchoring tumor immunity by increasing antigen presentation and chemokine production by tumor cells. Thus, the attenuation function of Eomes on IFN-γ during sustained T cell immune responses will have profound implication for tumor immunotherapy. Our recent study indicates that IL33 promotes antitumor immune responses by increasing tissue resident molecules (Chen et al., 2020). Eomes was shown to regulate CD103 expression in resident memory CD8^+^ T cells in a viral infection model (Mackay et al., 2015). Our study further demonstrated that Eomes inhibited tissue residency molecules in the setting of IL33 tumor therapy. This finding provides another mechanism by which Eomes limits tissue immune responses. Studies in models of chronic viral infections indicated TCF1^+^ CD8^+^ T cells can be hyper proliferative upon anti-PD-1 therapy and is critical for viral control and limiting tumor progression (He et al., 2016; Im et al., 2016; Leong et al., 2016; Wu et al., 2016; Kurtulus et al., 2019; Siddiqui et al., 2019). Another notable finding is that TCF1^+^ stem-like CD8^+^ TILs were increased in EKO mice. These data suggest Eomes is important for differentiation of stem-like CD8^+^ TILs into more terminal T cell fate. It is remarkable that Eomes deficiency led to a great increase in IFN-γ^+^ stem-like CD8^+^ T cells, indicating a critical role of Eomes in regulating the function of this dynamic T cell subset. Overall, Eomes plays a central role in shutting down the protracted T cell-mediated immune responses in the TME and such finding has significant implications in tumor immunotherapy.

In contrast to its function in limiting tissue T cell-mediated immune responses, Eomes promotes systemic tumor antigen-specific T cells. This is demonstrated by a decrease in central memory T cells in the secondary lymphoid system but an increase of these cells in the TME of EKO mice. This observation is consistent with the well-established role of Eomes in promoting central memory CD8^+^ T cells through upregulation of IL2rb (Intlekofer et al., 2005; Li et al., 2013). Consistent with a memory defect in EKO mice, we observed much reduced memory recall responses when mice were re-challenged with tumor cells at a distal site. The reduced antitumor activities to tumor growth at a secondary site in EKO mice were likely the combined effect of diminished memory T cells due to lower IL2rb and reduced tumor trafficking due to reduced expression of CXCR3 (Intlekofer et al., 2005; Li et al., 2013).

One intriguing finding in our study was that Eomes deficiency in T cells resulted in increased CD4^+^ T cells and Treg cells in the B16-IL33 tumors. Because we have shown that Eomes was minimally expressed in CD4^+^ T cells and Treg cells in tumors, it is quite unlikely that Emoes intrinsically regulated these cells. It is, however, possible that Eomes deficiency led to increased immunogenicity of the TME, which further increased CD4^+^ T cell-mediated antitumor immune responses. Such increased CD4^+^ and CD8^+^ T cell immune responses were counteracted by rising IL33-induced Treg cells. The exact mechanisms and the role of CD4^+^ T cells and Treg cells in tumor immunotherapy warrant future studies.

Taken together, our study has unified the biological role of Eomes in T cell-mediated antitumor immune responses by demonstrating that Eomes promotes resolution of T cell-driven tumor site immune responses and generation of systemic memory T cells. Our study demonstrates that local and systemic antitumor immunity are coordinately regulated through Eomes to balance local sterile tumor eradication and immune surveillance of metastatic dissemination. These findings should shed light on design and optimization of next generation T cell-based cancer immunotherapy.

## Materials and Methods

### Mouse

Eomes^*flox/flox*^ mice were originally obtained from Dr. Binfeng Lu at University of Pittsburgh. All mice are on the C57BL/6 background. CD4^*cre*^x Eomes^*flox/flox*^ mice were denoted as EKO mice. Both female and male mice were used and were aged 6–9 weeks at the time of the experiments. Mice were housed in the specific-pathogen-free (SPF) facility in the School of Medicine, Soochow University. All mouse experiments have been approved by institutional animal care and use committee of Soochow University.

### Cell Lines and Animal Models

Generation of B16-IL33 and B16-vec tumor cell lines were previously reported (Gao et al., 2015). B16 and B16-IL33 cells were cultured in RPMI medium 10% fetal bovine serum (FBS) and 1% penicillin-streptomycin (P.S). For the B16-vec or B16-IL33 tumor model, 0.1 million B16-vec or B16-IL33 cells were injected intradermally (i.d.) into the right flank of the mice. For the 3LL-vec or 3LL-IL33 tumor model, 0.2 million 3LL-vec or 3LL-IL33 cells were injected intradermally (i.d.) into the left flank of the mice. For tumor rechallenge experiment, 0.1 million B16 cells were injected intradermally (i.d.) into contralateral flank of the mice. MC38 cell line was cultured in DMEM medium 10% FBS and 1% P.S. For the MC38 tumor model, 1 million MC38 cells were injected intradermally (i.d.) into the right flank of the mice. PD-1 mAb (clone j43, catalog no. BP0033–2, BioXcell) was administered on the 5th day after the tumor inoculation. A total of 200 mg antibodies were intraperitoneally injected four times with 4-day intervals. Hamster IgG (catalog no. BE0091) was used as control. Tumor size was monitored and recorded every 2 days after tumor injection.

### Preparation of Single-Cell Suspension From Tumor Tissues

Tumor tissues were processed according to the protocol described before (Yang et al., 2020). In brief, mice were sacrificed, and tissues were freshly dissected. Tumor tissues were then cut into pieces and digested in serum free RPMI with 0.33 mg/ml DNase (sigma) and 0.25 mg/ml Liberase TL (Roche) and then grinded, washed in PBS, and filtered through a 70 μm cell strainer for single cell suspensions.

### Flow Cytometry

Flow cytometry experiments were all done by the instrument LSRII (BD) Aurora (Cytek Biosciences) and analyzed by Flowjo (BD). CD45 (clone 30-F11), CD4 (clone RMT4-5), CD8a (clone 53.67), Foxp3 (clone MF-14), Eomes (clone W17001A), T-bet (clone 4B10), IFN-γ (clone XMG1.2), GzmA (clone 3G8.5), GzmB (clone GB11), CD62L (clone MEL-14), CD44 (clone BJ18), PD-1 (clone J43), Tim3 (clone RMT3-23), Lag3 (clone C9B7W), CD69 (clone H1.2F3), and CD103 (clone 2E7) were purchased from BD Bioscience or Biolegend. TCF-1 (clone C63D9) was purchased from Cell Signaling Technology. For intracellular transcription factors and cytokines staining, cells were stimulated with leukocyte activation cocktail (BD) for 6 h and then followed the standard staining protocol described before (Yang et al., 2020).

### ELISpot Assay

15 μg/mL anti-IFN-γ (AN18, MabTech) was coated and incubated for 2 h at 37 degrees. Mice were sacrificed and splenocytes were isolated. 5 × 10^5^ splenocytes were co-incubated with 5 × 10^4^ 200Gy irradiated B16 tumor cells. 48 h later, plate was washed and incubated with 1.5 μg/mL biotinylated secondary antibody (R4-6A2, MabTech) and then washed and incubated with VECTASTAIN Elite ABC HRP Kit (Vector Labs) and developed with AEC Peroxidase (HRP) Substrate Kit (Vector Labs). Plate was further read and counted using the ImmunoSpot Analyzer (Cellular Technology).

### *In vitro* CD8^+^ T Cells Culture

Lymphocytes were collected from spleens and lymph nodes obtained from control (Eomes^*flox/flox*^) or EKO (CD4^*cre*^Eomes^*flox/flox*^) mice. Total CD8^+^ T cells were purified by negative selection (purity above 95%). The CD8^+^ T cells were cultured in Tc0 condition as indicated. Cells were stimulated with 5 μg/mL plate-bound anti-CD3 (clone 145-2C11) and 2.5 μg/mL plate-bound anti-CD28 mAbs (clone 37.51) in complete RPMI in the presence of hIL-2 (20 U/mL). After 48 h, cells were re-plated to new wells without anti-CD3 and anti-CD28 and with freshly added IL-2 (20 U/mL) for another 2 days. CD8^+^ T cells were washed with complete RPMI and were subsequently stimulated with TGF-β (1 ng/mL) for the induction of CD103 or with IL33 (10 ng/mL) plus IL-12 (3.4 ng/mL) for the induction of IFN-γ.

### Statistical Analysis

Statistical analysis was performed using Graphpad Prism v8. The log-rank test was used for comparisons in overall survival. Two-way ANOVA was used for comparing tumor growth curves. Two-tailed Student’s *t*-test was used for comparisons between two genotypes.

## Data Availability Statement

The original contributions presented in the study are included in the article/Supplementary Material, further inquiries can be directed to the corresponding author/s.

## Ethics Statement

The animal study was reviewed and approved by Soochow University, SYXK 2016-0050.

## Author Contributions

RS and YW devised and conducted experiments and analyzed data, wrote the manuscript. HZ, YW, YG, and JJ helped with the experiments. ZY helped import the mice. BL and YZ conceptualized and directed this study and wrote the manuscript. All authors contributed to the article and approved the submitted version.

## Conflict of Interest

The authors declare that the research was conducted in the absence of any commercial or financial relationships that could be construed as a potential conflict of interest.

## Supplementary Material

Supplementary Figure 1 Marker expression in TCF1^+^ and TCF1^–^ CD8^+^ TILs. **(A)** Representative flow cytometry plots showing Eomes co-stained with co-inhibitory molecules (PD-1, Tim-3, and Lag-3), effector molecules (IFN-γ, GzmB), resident T cells markers (CD69, CD103), resting marker TCF-1, and another T-box transcription factor T-bet in TCF-1^–^ tumor infiltrating CD8^+^ T cells. **(B)** Quantification of specific markers expression level in Eomes^–^ and Eomes^+^ CD8^+^ T cells depicted in panel **(A)**. **(C)** Representative flow cytometry plots showing Eomes co-stained with co-inhibitory molecules (PD-1, Tim-3, and Lag-3), effector molecules (IFN-γ, GzmB), resident T cells markers (CD69, CD103), resting marker TCF-1, and another T-box transcription factor T-bet in TCF-1^+^ tumor infiltrating CD8^+^ T cells. **(D)** Quantification of specific markers expression level in Eomes^–^ and Eomes^+^ CD8^+^ T cells depicted in panel **(C)**. **(E)** Representative flow cytometry plot showing the relationship between IFN-γ and granzyme B expression. **(F)** Representative flow cytometry plot showing Eomes co-stained with TNF-α in CD8^+^ T cells in B16-IL33 tumor. **(G)** Quantification of the percentage of Eomes positive CD8^+^ T cells between B16 and B16-IL33 tumors. **(H)** Quantification of the number of tumor-infiltrating CD8^+^ T cells between B16 and B16-IL33 tumors. Data were presented as mean ± SEM. ^∗∗^*P* < 0.01, Student’s *t*-test was performed.

Supplementary Figure 2 No significant difference in growth of B16 and 3LL tumors in control and EKO mice. **(A)** The B16 tumor cells (1 × 10^5^) were intradermally injected to control B6 or EKO B6 mice. Tumor sizes were monitored every 2 days, average sizes are shown. **(B)** Overall survival of B16 tumor bearing mice in control and EKO mice. **(C)** The 3LL tumor cells (2 × 10^5^) were intradermally injected to control B6 or EKO B6 mice. Tumor sizes were monitored every 2 days, average sizes are shown. **(D)** Overall survival of 3LL tumor bearing mice in control and EKO mice.

Supplementary Figure 3 Deletion of Eomes in T cells altered TME. **(A)** Representative flow cytometry plots showing GzmB and TCF-1 staining between control and EKO tumors. **(B)** Quantification of the percentage of GzmB^+^TCF-1^+^CD8^+^ T cells and GzmB^+^TCF-1^–^CD8^+^ T cells in CD8^+^ TILs. **(C)** Representative flow cytometry plots showing co-inhibitory receptors staining in TCF-1^–^ CD8^+^ T cells. **(D)** Quantification of the percent of PD-1^+^, Tim-3^+^, Lag-3^+^, and PD-1^+^Tim3^+^ CD8^+^ cells in the TCF-1^–^CD8^+^ T cells. **(E)** Representative flow cytometry plots showing co-inhibitory receptors staining in TCF-1^+^ CD8^+^ T cells. **(F)** Quantification of the percent of PD-1^+^, Tim-3^+^, Lag-3^+^, and PD-1^+^Tim3^+^ CD8^+^ cells in the TCF-1^+^CD8^+^ T cells. Data were presented as mean ± SEM. ^∗∗^*P* < 0.01, ^∗^*P* < 0.05, Student’s *t*-test was performed.

Supplementary Figure 4 Eomes directly regulates co-inhibitory receptors. Previously published Eomes-ChIPseq tracks (GSE122895), ATACseq tracks of tumor infiltrating CD8^+^ T cells (GSE110251) and *in vitro* cultured effector CD8^+^ T cells (GSE86797) were integrated in IGV genome browser at the PD-1, Tim-3, and Lag3 loci.
