# Supplementary material for: Eomes Impedes Durable Response to Tumor Immunotherapy by Inhibiting Stemness, Tissue Residency, and Promoting the Dysfunctional State of Intratumoral CD8+ T Cells
Source: Front Cell Dev Biol. 2021 Jan 21;9:640224. doi: 10.3389/fcell.2021.640224 (PMC7859102; doi:10.3389/fcell.2021.640224)
Supplement: Supplementary Figure 1 — Marker expression in TCF1+ and TCF1– CD8+ TILs. (A) Representative flow cytometry plots showing Eomes co-stained with co-inhibitory molecules (PD-1, Tim-3, and Lag-3), effector molecules (IFN-γ, GzmB), resident T cells markers (CD69, CD103), resting marker TCF-1, and another T-box transcription factor T-bet in TCF-1– tumor infiltrating CD8+ T cells. (B) Quantification of specific markers expression level in Eomes– and Eomes+ CD8+ T cells depicted in panel (A). (C) Representative flow cytometry plots showing Eomes co-stained with co-inhibitory molecules (PD-1, Tim-3, and Lag-3), effector molecules (IFN-γ, GzmB), resident T cells markers (CD69, CD103), resting marker TCF-1, and another T-box transcription factor T-bet in TCF-1+ tumor infiltrating CD8+ T cells. (D) Quantification of specific markers expression level in Eomes– and Eomes+ CD8+ T cells depicted in panel (C). (E) Representative flow cytometry plot showing the relationship between IFN-γ and granzyme B expression. (F) Representative flow cytometry plot showing Eomes co-stained with TNF-α in CD8+ T cells in B16-IL33 tumor. (G) Quantification of the percentage of Eomes positive CD8+ T cells between B16 and B16-IL33 tumors. (H) Quantification of the number of tumor-infiltrating CD8+ T cells between B16 and B16-IL33 tumors. Data were presented as mean ± SEM. ∗∗P < 0.01, Student’s t-test was performed. [file Data_Sheet_1.PDF]

Supplemental Figure Legend

Figure S1. Marker expression in TCF1$^+$ and TCF1$^-$ CD8$^+$ TILs. (A) Representative flow cytometry plots showing Eomes co-stained with co-inhibitory molecules (PD-1, Tim-3, and Lag-3), effector molecules (IFN-γ, GzmB), resident T cells markers (CD69, CD103), resting marker TCF-1, and another T-box transcription factor T-bet in TCF-1$^-$ tumor infiltrating CD8$^+$ T cells. (B) Quantification of specific markers expression level in Eomes$^-$ and Eomes$^+$ CD8$^+$ T cells depicted in (A). (C) Representative flow cytometry plots showing Eomes co-stained with co-inhibitory molecules (PD-1, Tim-3, and Lag-3), effector molecules (IFN-γ, GzmB), resident T cells markers (CD69, CD103), resting marker TCF-1, and another T-box transcription factor T-bet in TCF-1$^+$ tumor infiltrating CD8$^+$ T cells. (D) Quantification of specific markers expression level in Eomes$^-$ and Eomes$^+$ CD8$^+$ T cells depicted in (C). (E) Representative flow cytometry plot showing the relationship between IFN-γ and granzyme B expression. (F) Representative flow cytometry plot showing Eomes co-stained with TNF-α in CD8$^+$ T cells in B16-IL33 tumor. (G) Quantification of the percentage of Eomes positive CD8$^+$ T cells between B16 and B16-IL33 tumors. (H) Quantification of the number of tumor-infiltrating CD8$^+$ T cells between B16 and B16-IL33 tumors. Data were presented as mean ± SEM. **P< 0.01, Student's *t*-test was performed.

Figure S2. No significant difference in growth of B16 and 3LL tumors in control and EKO mice. (A) The B16 tumor cells($1x10^5$) were intradermally injected to control B6 or EKO B6 mice. Tumor sizes were monitored every two days, average sizes are shown. (B) Overall survival of B16 tumor bearing mice in control and EKO mice. (C) The 3LL tumor cells($2x10^5$) were intradermally injected to control B6 or EKO B6 mice. Tumor sizes were monitored every two days, average sizes are shown. (D) Overall survival of 3LL tumor bearing mice in control and EKO mice.

Figure S3. Deletion of Eomes in T cells altered TME. (A) Representative flow cytometry plots showing GzmB and TCF-1 staining between control and EKO tumors. (B) Quantification of the percentage of GzmB$^+$TCF-1$^+$CD8$^+$ T cells and GzmB$^+$TCF-1$^-$CD8$^+$ T cells in CD8$^+$ TILs. (C) Representative flow cytometry plots showing co-inhibitory receptors staining in TCF-1$^-$ CD8+ T cells. (D) Quantification of the percent of PD-1$^+$, Tim-3$^+$, Lag-3$^+$, and PD-1$^+$Tim3$^+$ CD8$^+$ cells in the TCF-1$^-$CD8$^+$ T cells. (E) Representative flow cytometry plots showing co-inhibitory receptors staining in TCF-1$^+$ CD8+ T cells. (F) Quantification of the percent of PD-1$^+$, Tim-3$^+$, Lag-3$^+$, and

PD-1^+Tim3^+ CD8^+ cells in the TCF-1^+CD8^+ T cells. Data were presented as mean ± SEM. $^{**}P<0.01$, $^{*}P<0.05$, Student's *t*-test was performed.

Figure S4. Eomes directly regulates co-inhibitory receptors. Previously published Eomes-ChIPseq tracks (GSE122895), ATACseq tracks of tumor infiltrating CD8^+ T cells (GSE110251) and in vitro cultured effector CD8^+ T cells (GSE86797) were integrated in IGV genome browser at the PD-1, Tim-3, and Lag3 loci.

Figure S1

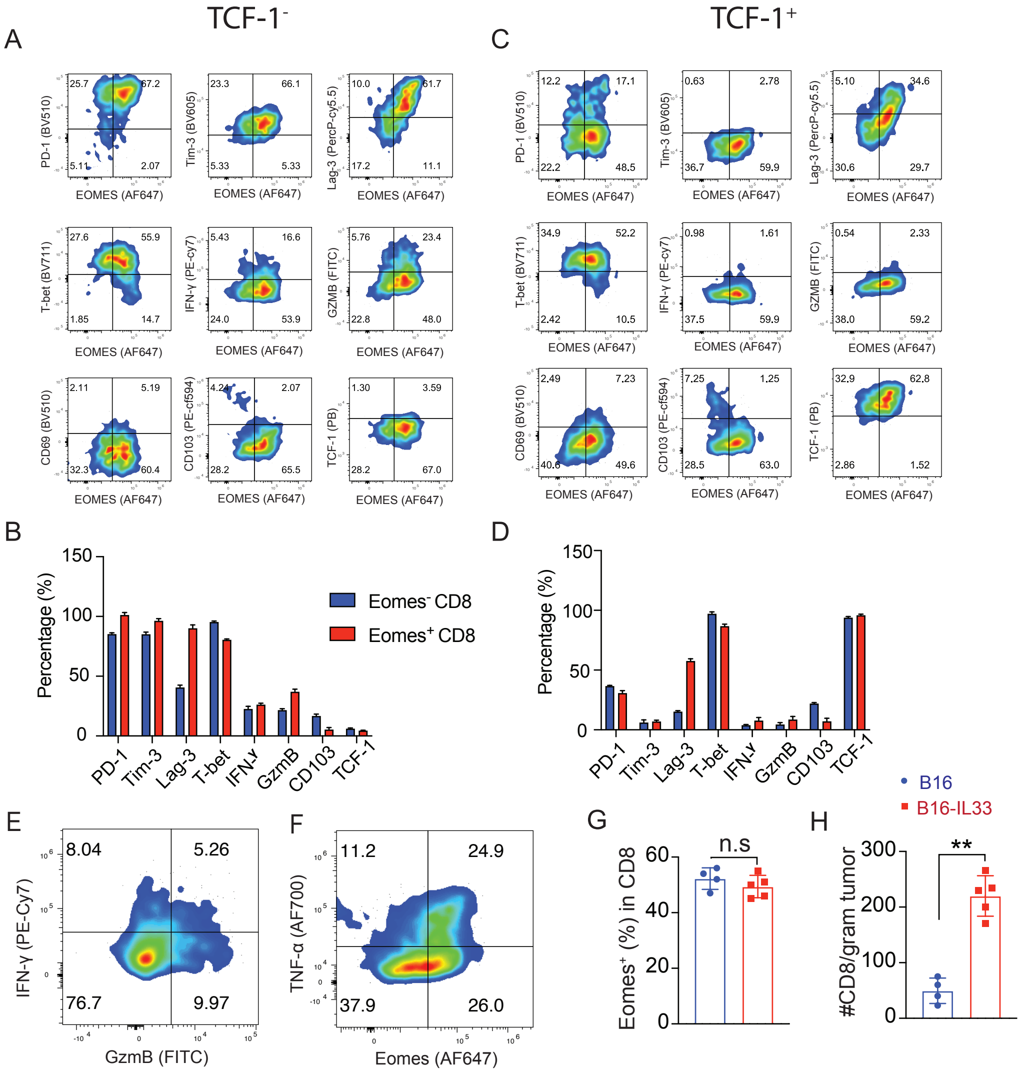

TCF-1⁻
TCF-1⁺
A
B
C
D
E
F
G
H
EOMES (AF647)
PD-1 (BV510)
Tim-3 (BV605)
Lag-3 (PercP-cy5.5)
T-bet (BV711)
IFN-γ (PE-cy7)
GZMB (FITC)
CD69 (BV510)
CD103 (PE-cf594)
TCF-1 (PB)
Percentage (%)
Eomes⁻ CD8
Eomes⁺ CD8
PD-1
Tim-3
Lag-3
T-bet
IFN-γ
GzmB
CD103
TCF-1
IFN-γ (PE-Cy7)
GzmB (FITC)
TNF-α (AF700)
Eomes (AF647)
Eomes⁺ (%) in CD8
#CD8/gram tumor
B16
B16-IL33
n.s
**

Figure S2

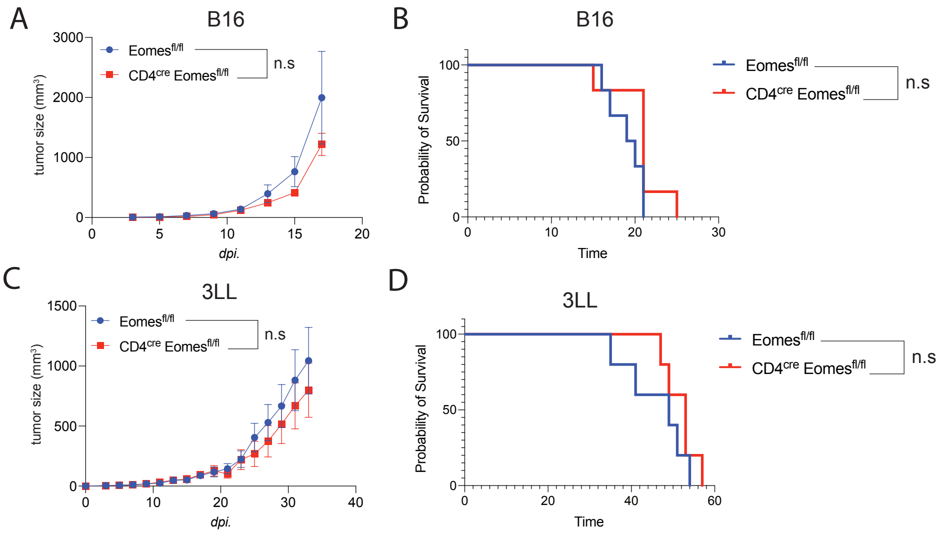
A
B16
3000
2000
1000
0
tumor size (mm3)
Eomesfl/fl
CD4cre Eomesfl/fl
n.s
0
5
10
15
20
dpi.
B
B16
Probability of Survival
100
50
0
0
10
20
30
Time
Eomesfl/fl
CD4cre Eomesfl/fl
n.s
C
3LL
1500
1000
500
0
tumor size (mm3)
Eomesfl/fl
CD4cre Eomesfl/fl
n.s
0
10
20
30
40
dpi.
D
3LL
Probability of Survival
100
50
0
0
20
40
60
Time
Eomesfl/fl
CD4cre Eomesfl/fl
n.s

Figure S3

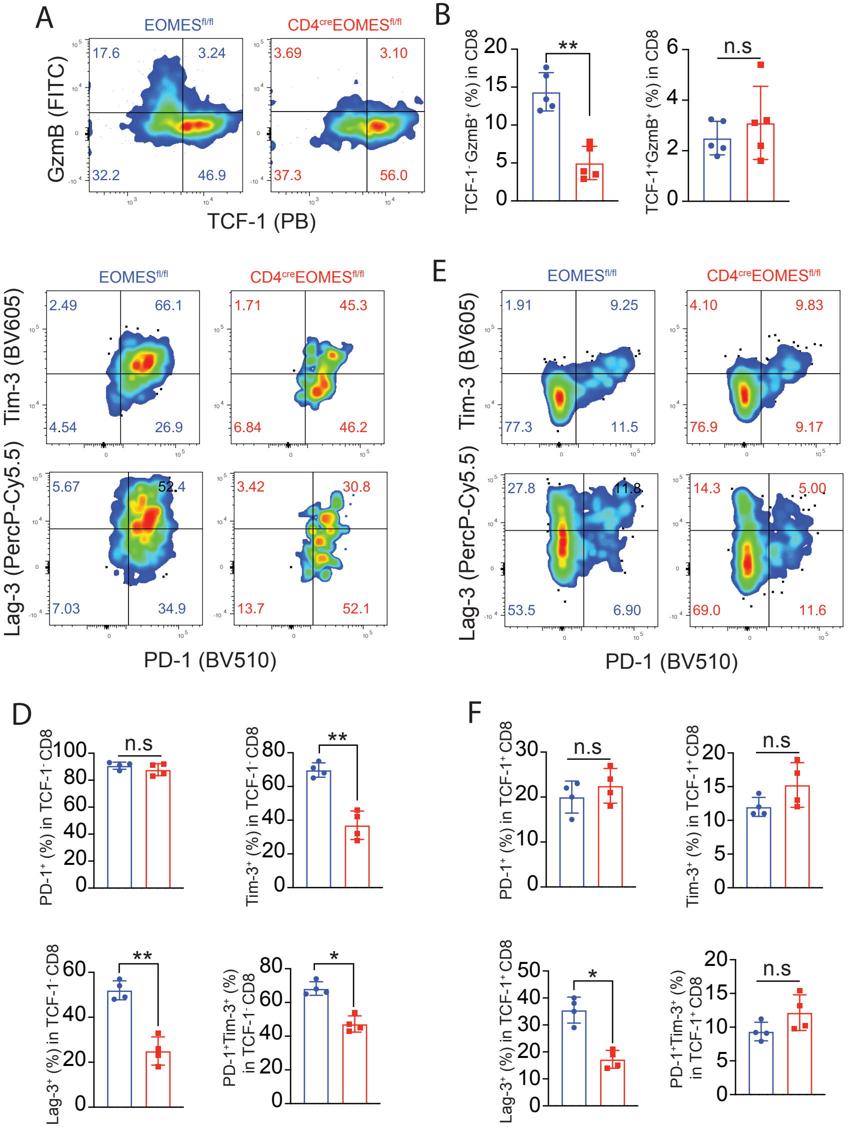
A
EOMES^fl/fl
CD4^cre EOMES^fl/fl
GzmB (FITC)
TCF-1 (PB)
17.6
3.24
32.2
46.9
3.69
3.10
37.3
56.0
B
TCF-1- GzmB+ (%) in CD8
TCF-1+GzmB+ (%) in CD8
**
n.s
C
Tim-3 (BV605)
Lag-3 (PercP-Cy5.5)
PD-1 (BV510)
2.49
66.1
4.54
26.9
1.71
45.3
6.84
46.2
5.67
52.4
7.03
34.9
3.42
30.8
13.7
52.1
E
1.91
9.25
77.3
11.5
4.10
9.83
76.9
9.17
27.8
11.8
53.5
6.90
14.3
5.00
69.0
11.6
D
PD-1+ (%) in TCF-1- CD8
Tim-3+ (%) in TCF-1- CD8
Lag-3+ (%) in TCF-1- CD8
PD-1+Tim-3+ (%) in TCF-1- CD8
F
PD-1+ (%) in TCF-1+ CD8
Tim-3+ (%) in TCF-1+ CD8
Lag-3+ (%) in TCF-1+ CD8
PD-1+Tim-3+ (%) in TCF-1+ CD8
*

Figure S4

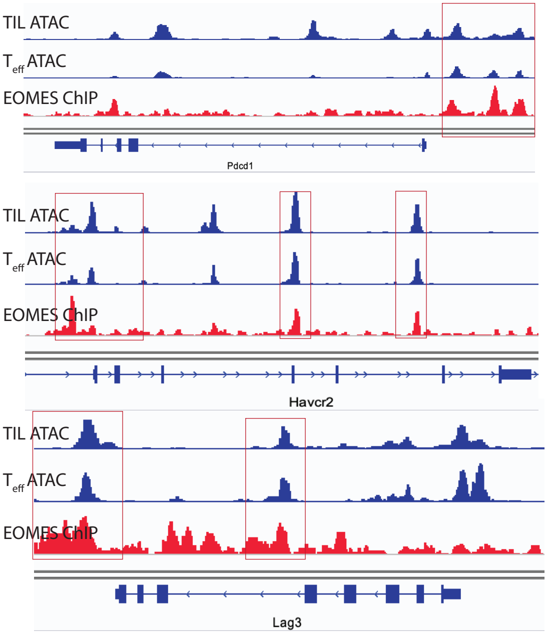
TIL ATAC
T_{eff} ATAC
EOMES ChIP
Pdcd1
TIL ATAC
T_{eff} ATAC
EOMES ChIP
Havcr2
TIL ATAC
T_{eff} ATAC
EOMES ChIP
Lag3
